# Characterising Shared and Specific Cell–Cell Communication in Cardiomyopathy Subtypes From Single‐Cell Transcriptomics Data

**DOI:** 10.1111/jcmm.70554

**Published:** 2025-05-08

**Authors:** Wenqi Tao, Miao Gong, Zunping Ke

**Affiliations:** ^1^ Department of Cardiology, Jing'an District Centre Hospital of Shanghai Fudan University Shanghai China; ^2^ Department of Geriatrics, Shanghai Fifth People's Hospital Fudan University Shanghai China

**Keywords:** cell–cell communications, diease specific intercellular interactions, molecular pathways, single nucleus RNA sequencing

## Abstract

Cardiomyopathy encompasses a diverse range of conditions characterised by extensive molecular heterogeneity, particularly the variations in cell–cell communication events such as ligand‐receptor interactions and downstream signalling. Understanding the common and unique features of these intercellular interactions is crucial for advancing targeted treatments. We analysed single‐cell RNA sequencing datasets from the ventricular regions of patients with arrhythmogenic cardiomyopathy (ACM), dilated cardiomyopathy (DCM), hypertrophic cardiomyopathy (HCM) and healthy donors (HD), as well as ischemic cardiomyopathy (ICM). Our analyses focused on cell type‐specific disease preferences, differential gene expression, pathway enrichment and particularly cell–cell communication. We observed that inflammatory, autoimmune, angiogenesis, lymphangiogenesis and fibrotic extracellular matrix deposition are consistently enriched across all four disease subtypes, highlighting their universal significance in disease progression through intercellular interactions. Additionally, we identified subtype‐specific pathways that reflect distinct intercellular communication patterns unique to each disease subtype: arrhythmia‐associated pathways in ACM, chronic inflammation‐related pathways in DCM, ECM remodelling pathways in HCM and ischaemic injury and recovery pathways in ICM.

## 1 Introduction

Cardiomyopathy is a spectrum of diseases where the heart muscle or its electrical system experiences structural and functional abnormalities characterised by significant heterogeneity at multiple levels. Such a spectrum encompasses a diverse set of diseases, with variability in cellular composition, gene pathways and intercellular communication, reflecting the various molecular mechanisms that drive the distinct progression of different disease subtypes. Cardiomyopathies can be categorised into primary types, originating from genetic, mixed, or acquired factors and secondary types, which are triggered by causes such as infiltration, toxins, or inflammation. The primary forms of cardiomyopathy include dilated, hypertrophic, restrictive, arrhythmogenic right ventricular cardiomyopathy and ischaemic cardiomyopathy [1].

Understanding the shared and unique molecular features across the cardiomyopathy spectrum is crucial for advancing treatment and potentially preventing the condition. Previous studies have shown that single‐nucleus RNA‐seq (snRNA‐seq) can effectively dissect transcriptomic heterogeneity, revealing differences in intercellular communication between healthy and diseased heart ventricular chambers across various disease subtypes. These findings have highlighted disease‐associated cell types and mechanisms of action (MoAs) such as ECM‐remodelled fibroblasts, angiogenesis and the activation of inflammatory signalling pathways [2, 3, 4, 5]. Moreover, mapping cell–cell communication using receptor‐ligand interaction databases has identified critical MoAs, including macrophage‐fibroblast interactions linked to impaired myocardial relaxation and increased myocardial stiffness [6, 7]. The snRNA‐seq datasets from multiple studies provide valuable insights that enable further comparison of disease‐specific cellular and intercellular communications among different disease subtypes, enabling a deeper understanding of both the shared and unique patterns of cellular and intercellular communications across the cardiomyopathy spectrum, which could inform more targeted therapeutic strategies.

In this study, we developed a comprehensive single‐cell reference using published snRNA‐seq datasets for ACM, DCM, HCM and ICM to explore the commonalities and distinctions in cellular landscapes and intercellular communication networks across various cardiomyopathy subtypes.

## 2 Materials and Methods

### 2.1 Data Sets

Single‐cell transcriptional profiling of ACM patients' data was from Reichart et al., 2022 (https://cellxgene.cziscience.com/collections/e75342a8‐0f3b‐4ec5‐8ee1245a23e0f7cb/private) [4]; DCM and HCM patients' data, as well as HD data, were obtained from Chaffin et al., 2022 (SCP1303 from Broad Institute Single Cell Portal) [2]; ICM patients' data were obtained from Simonson et al., 2023 (SCP1849 from Broad Institute Single Cell Portal) [5]. The processed files, including the expression matrix file (.mtx), the barcodes file (.tsv), the gene annotation file (.tsv) and the metadata file (.txt), will be used in our subsequent analysis.

We collected seven bulk RNA‐seq datasets from the GEO database, including samples from pre‐clinical mouse models, human cell lines and patient cohorts: GSE101301 (mouse model, ACM), GSE106201 (mouse model, DCM), GSE112055 (mouse model, HCM), GSE129134 (mouse model, ICM), GSE289628 (human cell line model, ACM), GSE116250 (patients, DCM & ICM) and GSE89714 (patients, HCM). Gene expression read counts from each dataset were downloaded and used for downstream analyses.

### 2.2 Processing of Single‐Cell RNA Seq Data Sets

The Seurat object for each patient dataset was created using Seurat (5.1.0). Most downstream analyses were also conducted using Seurat (5.1.0) [8]. We used quality control settings to exclude cells with fewer than 200 unique genes, or 5% or higher mitochondrial gene content. In addition, genes expressed in fewer than 3 cells were excluded. Next, we selected samples for each patient group, ensuring comparable cell numbers between samples within the group. Matrices were scaled and normalised using the ‘sctransform’ function in Seurat. Samples from three different datasets were then merged, retaining only overlapping feature genes. PCA was performed using 30 principal components. Batch correction using Harmony [9]. Cellular identities were assigned using the ‘celltype.l1’ and ‘celltype.l2’ annotations, which are derived from integrated human heart data. These references have been incorporated into the annotated reference dataset in Azimuth [10, 11, 12, 13].

### 2.3 Differential Gene Expression (DGE) Analysis

DGE analysis between disease and healthy samples within the same cell type for each disease subtype was conducted following the ‘de_vignette.Rmd’ from the Seurat vignettes (https://github.com/satijalab/seurat.git). DGE analysis was performed at both the single‐cell and pseudobulk levels. Upregulated DGEs were identified as genes that were significantly upregulated (*p*_value < 0.05 and log2FC > 1) in both analyses. We further selected uniquely expressed genes and the top 10 upregulated genes for each cell type and disease subtype to visualise their expression levels in a heatmap.

### 2.4 Gene Set Enrichment Analysis (GSEA) and Single Sample Gene Set Enrichment Analysis (ssGSEA)

We conducted GSEA and ssGSEA analyses using the GSVA R package following the provided guidelines [14]. The analysis included gene sets from the Molecular Signatures Database (MSigDB) version 7.1, covering the Hallmark Collection and Canonical Pathways (Reactome).

### 2.5 Cell–Cell Communication

The MultiNicheNet package (version 2.0.0) was used to assess interactions between cell clusters based on ligand‐receptor information within signalling pathways [15]. The conditions were defined by four disease subtypes and one healthy group, with each disease condition compared to the healthy condition to identify specific cell–cell communication patterns. We followed the default MultiNicheNet pipeline as described in the ‘vignettes/paired_analysis_SCC.knit.md’ from the multinichenetr repository (https://github.com/saeyslab/multinichenetr.git). For each patient/healthy donor sample, disease/healthy group and annotated cell type, the identifiers were defined as ‘sample_id’, ‘group_id’ and ‘celltype_id’, respectively, when initiating the analysis. To identify shared ligand‐receptor (L‐R) pairs, the top 100 L‐R pairs for each disease were selected and compared to determine conserved pairs. For disease‐specific L‐R pairs, the top 250 L‐R pairs for each disease were selected and compared to identify unique pairs for each disease subtype.

## 3 Results

### 3.1 Integration of Single‐Nucleus RNA‐Sequencing Data Across Cardiomyopathy Subtypes and Healthy Controls

Characterising the compositional and cell‐type‐specific signalling characteristics of cardiovascular diseases is crucial for advancing our understanding of their role in the heterogeneous development of various cardiomyopathy subtypes. To address this, we constructed a comprehensive single‐cell reference, assembled using published studies from patient cohorts, to investigate the differences in complex disease modulation processes between cardiomyopathy disease subtypes.

**FIGURE 1:**
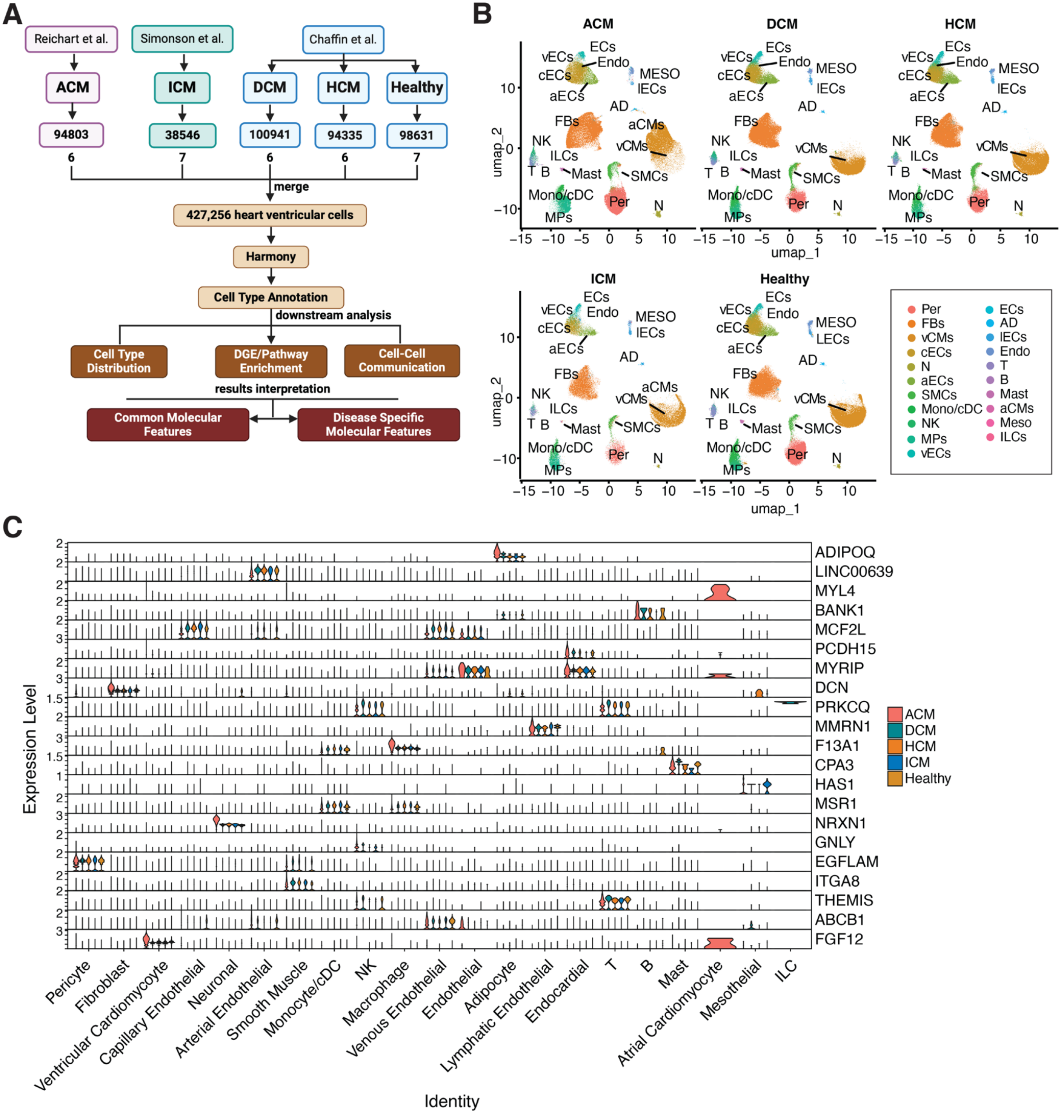
Identification of Major Cell Compartments in Human Cardiomyopathy and Healthy Donors through Single‐cell Data Integration. (A) Overview of Dataset Integration, Downstream Analysis and Interpretation of Results Using Single‐Cell RNA Sequencing Datasets from Three Independent Studies. (B) UMAP embeddings of ‘celltype.l2’ cell types, split by disease subtype and healthy donor group. Cell types are labelled by abbreviation as follows: Adipocyte (AD), Arterial Endothelial cells (aECs), Atrial Cardiomyocyte cells (aCMs), B cells (B), Capillary Endothelial cells (cECs), Endocardial (Endo), Endothelial cells (ECs), Fibroblasts (FBs), Innate Lymphoid cells (ILCs), Lymphatic Endothelial cells (lECs), Macrophages (MPs), Mast (Mast), Mesothelial cells (MESO), Monocyte/cDC (Mono/cDC), Neuronal cells (N), NK/T cells (NK), Pericytes (Per), Smooth Muscle cells (SMCs), T cells (T), Venous Endothelial cells (vECs) and Ventricular Cardiomyocyte cells (vCMs). (C) Stack violin plots showing the ‘celltype.l2’ annotated cell types specific expression of the top‐ranking candidate marker genes for each identified cell type across disease subtype and healthy donor group.

To ensure equitable representation of cell types and sequencing depth, we analysed 427,256 single‐cell transcriptomes from 6 DCM patients, 6 HCM patients, 6 ACM patients, 7 ICM patients and 7 healthy donors, ensuring a comparable number of cells. Rigorous quality control, filtering and batch correction were then conducted. Samples subsequently underwent unsupervised graph‐based clustering (Figure 1A). Uniform Manifold Approximation and Projection for Dimension Reduction (UMAP) revealed that the heart transcriptomes from 32 individuals aggregated in 16 clusters (0–15) (Figure S1A). Meanwhile, we utilised Azimuth with its validated reference datasets to characterise the cell identities within these clusters [11, 12, 13]. In comparing the inferred cell types from Level_1 (‘celltype.l1’) and Level_2 (‘celltype.l2’), two reference atlas from Azimuth, we observed a high consistency between the levels. For instance, cells classified as “Cardiomyocyte” in Level_1 were further refined into “Atrial Cardiomyocyte” and “Ventricular Cardiomyocyte” in Level_2, maintaining the overarching classification while providing additional specificity. Similarly, the broad category of ‘Myeloid’ in Level_1 was consistently divided into ‘Macrophage’ and ‘Monocyte/cDC’ in Level_2, reflecting the expected biological distinctions (Figure S1A). This alignment across levels underscores the reliability of the cell type predictions and reinforces the hierarchical nature of the annotations.

The batch‐grouped UMAP plots following batch correction displayed a marked reduction in batch effects. Cells from different batches were overlayed, forming coherent clusters based on cell type rather than batch origin, confirming the reliability of our cell type annotations for subsequent analysis (Figure S1B). Also, we conducted UMAP visualisation of the data before and after batch correction to evaluate the impact of batch effects on cell type annotation. The consistent UMAP patterns across different batches imply that our results are not biased by batch‐specific variations. The UMAP plots split by disease subtype revealed similar patterns across all subtypes and the healthy donor group, suggesting that the cell‐type spectrum in heart tissue is relatively conserved among these groups (Figure S1C). The cell types with their specific markers, selected from the ‘Level_1’ dataset, included Adipocyte (AD; CIDEA, ADIPOQ, PLIN+), B (B; FCRL5+), Cardiomyocyte (CM; RYR2, MLIP, MYOM3+), Endothelial (EC; ID1, ADGRL4, VWF+), Fibroblast (FB; MFAP5, MEG8, ACSM3+), Lymphatic Endothelial (LEC; CCL21, TBX1, MMRN1+), Mast (RAB44, CPA3, KIT+), Mesothelial (MESO; ITLN1, PRG4+), Myeloid (ML; FCGR2A, F13A, CD163+), Neuronal (N; ST6GALNAC5, XCR4, NRXN1+), NK/T (PRF1, NKG7, CD247+), Pericyte (PER; RGS5, EGFLAM, GUCY1A2+) and Smooth Muscle (SMC; SUSD5, LGR6, MYH11+). We observed that some marker genes exhibit a conserved high expression pattern across all disease subtypes; there are also marker genes whose expression varies inconsistently between different disease subtypes (Figure S1D).

To better elucidate cellular diversity and enhance cell annotation precision, we incorporated Level_2 to Level_1. This inference provided a finer resolution, refining the initial 13 cell types to 21 distinct cell types in our transcriptome data cohort and offering more detailed insights into cell populations (Figure 1B). These annotations were supported by the expression of well‐established marker genes in corresponding Level_2 cell types (Figure 1C).

### 3.2 Assessing Annotated Cell Type Heterogeneity in Disease Subtypes

**FIGURE 2:**
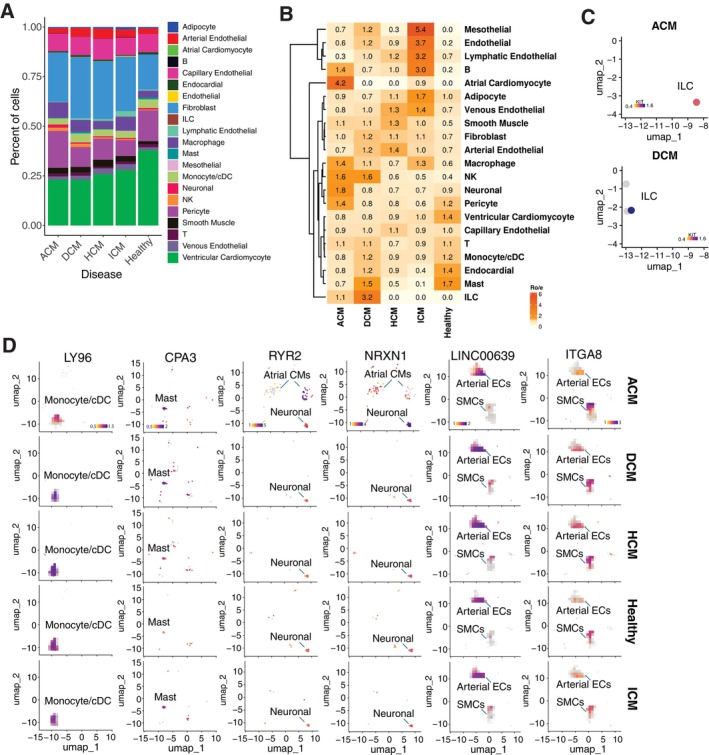
Characterise Cell Composition in Each Patient Subgroup and Evaluate Cell Type‐Specific Disease Preferences. (A) Stack bar plots showing the distribution of Level_2 annotated cell types across disease subtypes and the healthy group. (B) Heatmap showing disease preference for each annotated cell type, evaluated by the ratio of observed to expected cell counts (Ro/e) value. (C) UMAP plot showing the expression patterns of the ILC marker gene KIT in annotated ILC cells across detected patient groups. (D) UMAP plot showing the expression patterns of the genes, including LY96(MD‐2), CPA3, NRXN1, RYR2, LINC00639 and ITGA8 in annotated Monocyte/cDC, Mast, Atrial Cardiomyocyte, Neuronal, Arterial Endothelial and Smooth Muscle, respectively. Split by disease subtype and healthy donor group. Dot colours represent expression level.

Based on this high‐resolution cell type annotation, we observed a balanced proportion of capillary endothelial cells across all groups. In contrast, other cell types showed notable variability in proportions between patient groups with different disease subtypes and between these subtypes and the healthy donor group (Figure 2A). To further assess the cardiomyopathy disease subtype distribution preference of annotated cell types with statistical power, we calculated the ratio of observed to expected (Ro/e) cell numbers for each annotated cell type in different patient groups as well as the healthy donor group, where the expected cell numbers of cell types in a given group were derived from the chi‐square test. Through this analysis, Atrial Cardiomyocyte (aCM) and Neuronal (N) cells showed the strongest preference in ACM; Innate Lymphoid Cells (ILCs) were most enriched in DCM; Capillary Endothelial Cells (cECs) were slightly enriched in HCM; and Ventricular Cardiomyocyte (vCM) was enriched in HD (Figure 2B). Additionally, Mesothelial (MESO), endothelial (including lymphatic, arterial and venous types), B, Adipocyte (AD), Smooth Muscle Cells (SMCs), Fibroblasts (FBs), Macrophages (MPs) and NK cells demonstrated enrichment in more than one patient group, but not in the HD, suggesting their potential involvement in disease progression (Figure 2B).

ILCs are implicated in DCM pathogenesis, potentially promoting the disease through cytokine overproduction [16, 17]. Despite a high Ro/e score, only 4 ILCs were identified in the dataset with inconsistent marker gene expression (Figure 2C). This suggests that the score may not accurately reflect ILCs' abundance or function in DCM, warranting further investigation. Aside from ILCs, we found that Monocytes/cDC (Mono/cDC) cells, Mast cells and NK cells were more highly enriched in the DCM subgroup compared to other disease subgroups (Figure 2B). Specifically, we observed elevated levels of MD‐2 and CD56 in a significant portion of the Mono/cDC cell population in DCM (Figure 2D), which are known to facilitate monocyte recruitment and play a crucial role in DCM progression [18]. Additionally, Mast cells, identified by CPA3, were also enriched in DCM (Figure 2D), potentially influencing the activation of myofibroblasts [19]. These findings collectively suggest that these immune cells may play important roles in DCM, as supported by existing studies.

We also observed that all four types of endothelial cells—capillary, lymphatic, arterial and venous—along with smooth muscle cells, are enriched in HCM (Figure 2B,D). These findings are consistent with established knowledge that endothelial cells and smooth muscle cells are crucial for vascular remodelling, angiogenesis and heart fibrosis, and that alterations in these cells can significantly influence the progression of HCM [20, 21].

Neuronal cells (labelled by NRXN1) and aCM cells (labelled by RYR2) are both enriched in ACM within our integrated single‐cell transcriptome cohort (Figure 2B,D). The robustness and specificity of the enrichment of these two cell types in ACM are closely linked to their mechanism of action in disease development. The adipose‐neural axis plays a critical role in cardiac arrhythmias [22]; while aCM expresses inflammatory signalling pathways that can contribute to atrial fibrillation (AF) [23].

Compared to the other three NICM types, the cell‐type spectrum enriched in ICM is more diverse, including mesothelial cells, adipocytes, various endothelial cell types as well as innate and adaptive immune cells (Figure 2B). This diversity suggests that the progression of ICM is influenced by a complex interplay of molecular and cellular factors, such as fibrosis, scarring, endothelial dysfunction and inflammatory responses, highlighting the multifaceted nature of the disease's pathology [24, 25, 26, 27].

### 3.3 Characterise Gene Expression and Pathway Changes for Each Cell Type Across Cardiomyopathy Disease Subtypes

**FIGURE 3:**
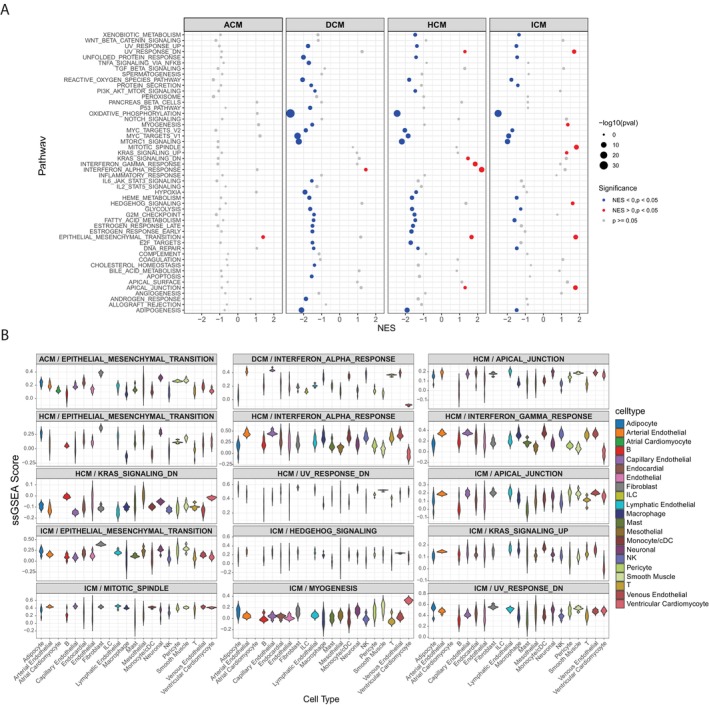
Gene Set Enrichment Analysis Reveals Hallmark Gene Sets Across Cardiomyopathy Subtypes and Annotated Cell Types. (A) Dot plot showing Hallmark gene‐set enrichment analysis comparing disease versus healthy controls across four cardiomyopathy subtypes (ACM, DCM, HCM, ICM). The plot is faceted by disease subtype, with the x‐axis representing the normalised enrichment score (NES) and the y‐axis indicating pathway names. Dot size corresponds to the −log10 (*p*‐value), and dot colour indicates statistical significance. (B) Violin plot showing single‐sample gene set enrichment analysis (ssGSEA) scores for pathways identified in Panel A across annotated cell types. The plot is faceted by enriched pathways from each disease subtype, with the *x*‐axis and violin colour representing the annotated cell types and the *y*‐axis indicating the ssGSEA score.

To explore shared and distinct transcriptional changes between disease and healthy controls for each cardiomyopathy subtype, we conducted differential gene expression (DGE) analysis across all annotated cell types by aggregating nuclei across samples into a pseudo‐bulk representation. In the meantime, we performed DGE analysis on a single‐cell level. The resultant gene lists from pseudo‐bulk and single‐cell DGE analyses were merged, and cell types that did not show up‐regulated genes were filtered out (log2FC > 1, *p*‐value < 0.05), as described in Section 2 (see Table S1 for details). Next, we aim to identify gene signatures enriched in disease compared to healthy controls, focusing on pathways relevant to cardiomyopathy progression across the four disease subtypes. Disease‐enriched pathways were identified using gene set enrichment analysis (GSEA), while single‐sample gene set enrichment analysis (ssGSEA) was used to evaluate the enrichment of these pathways across annotated cell types. Both analyses applied Hallmark and KEGG gene sets (Figure 3A,B and Figure S2A,B, also described in Section 2).

We observed that the epithelial‐mesenchymal transition (EMT) signature was enriched in three subtypes: ACM, HCM and ICM, while the apical junction signature was enriched in both HCM and ICM (Figure 3A). Additionally, signatures of focal adhesion and extra‐cellular matrix (ECM) receptor interaction were enriched in DCM, HCM and ICM (Figure S2A). These findings suggest that both cell–cell and cell‐matrix interactions are conserved and critical for disease progression. In terms of the enrichment of these signatures across annotated cell types, we found that the EMT signature was enriched in fibroblast cells, while the apical junction signature was enriched in lymphatic endothelial cells (Figure 3B). The signatures of focal adhesion and ECM receptor interaction were enriched in fibroblasts, smooth muscle cells, neuronal cells and lymphatic endothelial cells (Figure S2B) These findings suggest that these cell‐signalling pathways may be connected to cardiac or cardiovascular dysfunction and could be associated with the underlying fibrosis and ECM remodelling in the respective diseases. Additionally, we observed that the interferon alpha response signature was conserved across DCM and HCM and was enriched in endothelial cells, including arterial endothelial cells, capillary endothelial cells and venous endothelial cells (Figure 3A,B). In the context of two disease subtypes, interferon alpha (IFN‐α) can harm endothelial cells, compromising their function and potentially accelerating disease progression. This may occur through mechanisms such as decreased nitric oxide synthesis, suppression of angiogenesis and heightened inflammation in the cardiac vasculature. Ultimately, IFN‐α can disturb the delicate balance required for adequate blood flow in the impaired heart muscle.

We then observed several signatures uniquely enriched in ICM, indicating that the ICM sample displayed significant transcriptomic differences compared to the other three NICM samples. The identified pathways include myogenesis, mitotic spindle and hedgehog signalling (Figure 3A). Myogenesis was enriched in smooth muscle cells and ventricular cardiomyocytes. The results also suggest that both the mitotic spindle and hedgehog signalling pathways are activated across a variety of cell types in ICM. Specifically, these pathways were found to be enriched in fibroblasts, macrophages, Mono/cDCs, smooth muscle cells, pericytes, ventricular cardiomyocytes and endothelial cells (Figure 3B). This indicates that these pathways may play a role in the cellular processes associated with ischaemic injury and repair in the heart. For example, the mitotic spindle is involved in cell division, which may be crucial for tissue regeneration, while hedgehog signalling is often linked to developmental processes and tissue repair, potentially influencing the behaviour of these cell types during ischaemic damage [28]. These findings suggest that these signalling pathways are broadly active in the heart's response to ischaemia, highlighting their potential role in modulating inflammation, fibrosis and tissue remodelling.

### 3.4 Analyse Altered Cell–Cell Communication Between Disease and Healthy States for Each Cardiomyopathy Subtype

We inferred and prioritised disease‐specific L‐R pairs by comparing disease and healthy groups for each disease subtype of ACM, DCM, HCM and ICM. These pairs were quantified using prioritisation scores based on cell‐type specificity, higher expression in disease compared to healthy control, predominant presence in the disease of interest and enrichment of predicted target genes in the receiving cell types. By analysing the ranks of these pairs according to their prioritisation scores, we inferred intercellular signalling and communication, allowing us to investigate differences in cell–cell communications between diseased and healthy states using Multinichenetr [15, 33].

**FIGURE 4:**
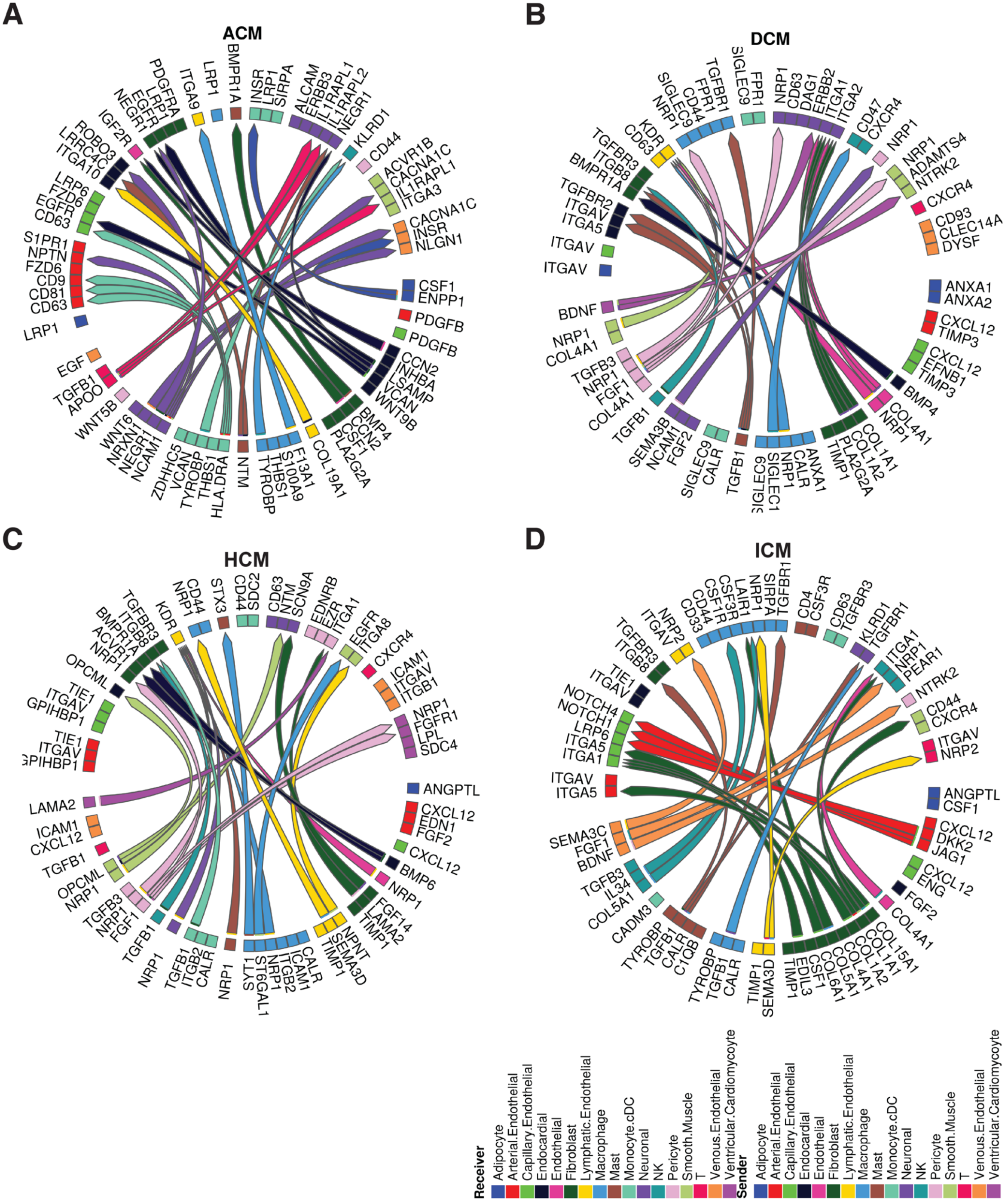
Summary of cell–cell communication in patients. Interactome analysis was performed on the annotated scRNAseq analysis of disease vs. healthy control using the MultiNicheNet package. Circos plots showing the top 30 ligand‐receptor interactions differentially activated in disease subtypes compared to healthy controls: ACM (A), DCM (B), HCM (C) and ICM (D). All clusters listed in the sender and receiver columns within the respective disease group of the prioritised table were included.

We noted that these cell–cell interactions were widespread and evident in most cell types, including various immune cells, multiple vascular endothelial cells and several types of cardiomyocytes across each cardiomyopathy. In ACM, there are 13 sender and 15 receiver cell types (Figure 4A); in DCM, 14 sender and 13 receiver cell types (Figure 4B); in HCM, 17 sender and 14 receiver cell types (Figure 4C); and in ICM, 12 sender and 13 receiver cell types (Figure 4D).

A more focused analysis of immune cell subtypes and their interactions with other cardiac cell types, based on cell abundance, was conducted by selecting the top two immune cells and top two non‐immune cardiac cells. This approach aims to provide valuable insights into the roles of these cells and their contributions to the development and progression of cardiomyopathies (Figure S3).

The top interactions in ACM include L‐R pairs of PROS1‐MERTK, PROS1‐MERTK and APOO‐IL1RAPL1, indicating the interplay between cardiac remodelling and inflammation response (Figure S3A). In DCM, we observed frequent interactions involving TGFB1 with ITGB8, as well as IGSF10 with MILR1/CD200R1 (Figure S3B). Interestingly, the TGFB1 signalling was consistently found in HCM, suggesting shared mechanisms in disease progression (Figure S3C).

In HCM, the PDGFB‐PDGFRA and ADAM10‐EphA3 interactions suggest involvement in angiogenesis and tissue remodelling (Figure S3C). In the ICM patient group, the TGFB2‐TGFBR1 interaction between fibroblasts and macrophages was observed (Figure S3D). Interestingly, we also identified an interaction between TGFB1 from macrophages and ITGB1 from fibroblasts in HCM (Figure S3C). While both cell types are known to produce TGF‐beta, our findings further support the role of fibroblast‐derived TGFB2 in promoting fibrosis, inflammation and impaired tissue repair, contributing to the progression of ischemic cardiomyopathy [35].

The ligand‐receptor interactions identified through the above analysis further validate that immune/inflammatory signalling pathways, fibrosis, myocardial/ECM remodelling and angiogenesis are relatively conserved and shared across the four representative cardiomyopathies studied [34]. These interactions could be considered potential therapeutic targets.

## 4 Discussion

Cardiomyopathy encompasses a diverse range of heart muscle disorders, including HCM, DCM, ACM and ICM [36]. Each type varies in its severity and impact on heart function, from increased wall thickness and impaired contractility to arrhythmias, ischemic damage and heart failure. Various cell types are involved in these conditions, including cardiomyocytes, which are directly affected, as well as fibroblasts, vascular smooth muscle cells (VSMCs) and endothelial cells [37, 38]. Recent research also highlights the role of immune cells, such as MPs, in modulating inflammation and fibrosis. These cells contribute to the structural and functional alterations observed in the disease [39]. To better understand these disease‐specific alterations, snRNA‐seq datasets from patients' ventricular regions are invaluable for identifying dysregulated cell types and transcriptional pathways associated with each cardiomyopathy subtype.

Within the integrated snRNA transcriptome profile that includes four disease subtypes, we assessed the disease‐specific distribution of each identified cell type after finding no significant differences in overall cell type composition (Figure 2A,B). Our results largely replicate those of the original studies. For example, both angiogenic and lymphangiogenic endothelial cells are enriched in ICM [5]. There is relatively little difference between DCM and HCM, suggesting a closer transcriptional profile between the two [2]. We also acknowledge a limitation in our study: the cell state changes primarily examined in the original studies were not included in our results, as they were not part of our analysis [4].

Our pathway analysis, based on gene set enrichment analysis for each disease subtype within each cell type, focused on identifying the shared and distinct gene pathways among the four disease subtypes. Our findings indicate that the top shared pathways of EMT are predominantly concentrated in FBs (Figure 3A,B). In FBs, we identified pathways associated with cytoskeleton and ECM‐related signalling, suggesting fibroblast remodelling is a common cellular mechanism in disease progression (Figure S2A,B). This finding aligns with observations from the original study [4].

Intercellular communication is crucial for maintaining homeostasis in the heart and for cardiac repair after injury. However, studies have shown that this communication is altered in different types of cardiomyopathies, playing a pivotal role in disease progression. Our analysis focused on characterising disease‐specific ligand‐receptor‐target communication patterns for each cardiomyopathy subtype and uncovered potential essential intercellular signalling pathways. This was achieved using MultiNicheNet, a recently developed tool for differential cell–cell communication analysis from multi‐sample, multi‐condition snRNA‐seq data. A meaningful interpretation of the results is that by comparing the differences in the same molecular interactions across different diseases, we can explore how signalling function links to cell communications and the pathogenesis of each disease.

**FIGURE 5:**
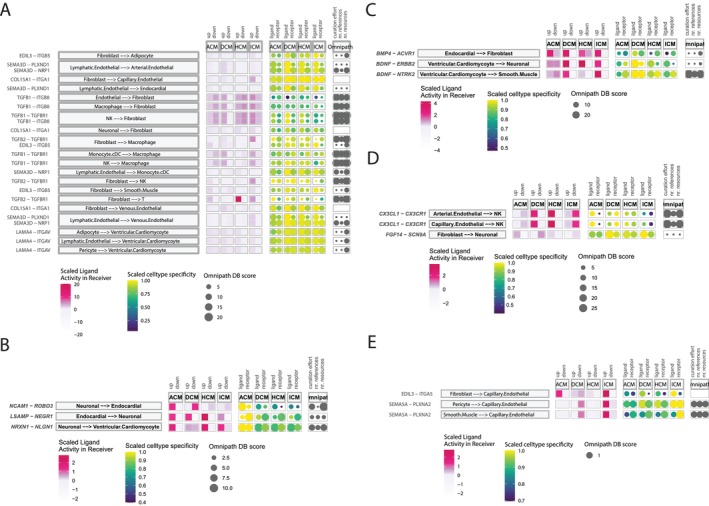
Summary of Shared and Disease‐Specific Cell–Cell Communications Across Disease Subtypes. Bubble plots display ligand‐receptor pairs and their differential impact across disease subtypes: (A) shared across three or more disease subtypes, (B) preferentially affected in ACM, (C) preferentially affected in DCM, (D) preferentially affected in HCM and (E) preferentially affected in ICM. Each plot includes scaled ligand activity in receiver cells, scaled cell type activity and curation levels of intercellular communication based on the Omnipath dataset.

As shown in Figure 5A, we identified 8 L‐R pairs—COL15A1‐ITGA1, EDIL3‐ITGB5, LAMA4‐ITGAV, SEMA3D‐NRP1, SEMA3D‐PLXND1, TGFB1‐ITGB8, TGFB1‐TGFBR1 and TGFB2‐TGFBR1—that are conserved across at least three of the four diseases. Among these, the ligand of SEMA3D from the lymphatic endothelial cells was the top incident, suggesting the prevalent involvement of vascular abnormalities in disease progression [29, 30, 31, 32, 40, 41]. Two TGF‐β isoforms, TGFB1 and TGFB2, were the most prevalent ligands observed across five sender cell types—endothelial cells, MPs, Mono/cDCs, NK cells and FBs—suggesting a broad role for TGF‐β signalling in disease progression. This signalling may influence tissue development, wound healing, cell differentiation and immune regulation. Given that dysregulated TGF‐β signalling is linked to tissue fibrosis and inflammatory diseases, these findings highlight its potential involvement in pathological processes. We further observed that integrin, a multifunctional family of molecules, plays a key role in ECM remodelling, fibrosis and inflammatory response. These receptors are prevalent across seven receiver cell types, including AD, various endothelial cells, FBs, MPs, SMCs and vCMs. The interaction between integrins and TGF‐β signalling is critical for regulating tissue homeostasis, fibrosis and inflammation through various mechanisms. Together, integrin‐TGF‐β crosstalk enables immune cells to modulate fibroblast behaviour, while fibroblasts influence the immune microenvironment, contributing to both tissue repair and pathological remodelling in cardiomyopathy [42].

For the ligand‐receptor interactions specific to each disease subtype. Among the interactions specific to ACM, most are inferred to occur between neurons and myocardial cells, suggesting a role for these interactions in arrhythmia‐associated disease progression (Figure 5B). For instance, preclinical and clinical studies have indicated that sympathetic neurons significantly drive myocyte remodelling in cardiomyopathy. The removal of sympathetic nerves has been shown to alleviate arrhythmias and reduce the incidence of sudden cardiac death. Additionally, the interaction of NRXN1‐NLGN1 has been reported to be associated with both epilepsy and cardiac arrhythmia (Figure 5B) [43]. DCM‐specific interactions, including BMP4‐ACVR1 and BDNF‐NTRK2 (Figure 5C), may collectively reflect the pivotal roles of inflammation—whether resulting from pathogenic infections or autoimmune signalling—in myocarditis, which subsequently leads to DCM [44, 45]. HCM‐specific interactions, such as FGF14‐SCN9A, may play significant roles in ECM remodelling, a hallmark of HCM. Additionally, the CX3CL1‐CX3CR1 interaction may be linked to chronic inflammation associated with hypertrophy (Figure 5D) [46]. The ICM‐specific interaction, SEMA5A‐PLXNA2, is involved in tissue remodelling (Figure 5E). These processes are associated with ischemic injury and recovery, highlighting potential implications for therapeutic strategies aimed at promoting tissue repair and regeneration.

**FIGURE 6:**
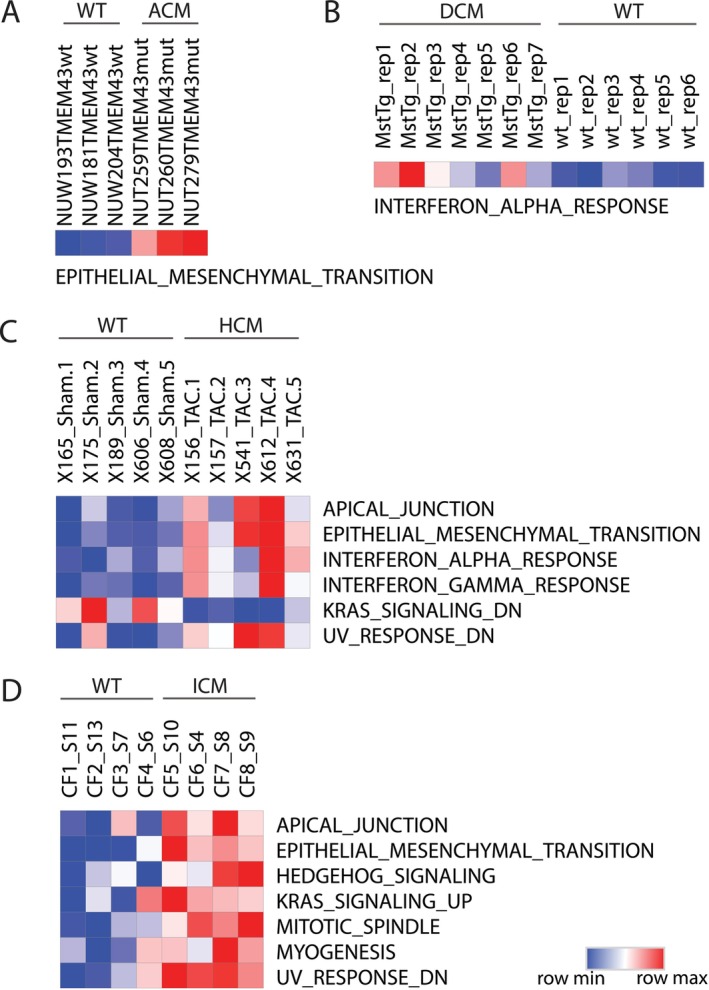
Validation of Disease‐Enriched Pathways in Cardiomyopathy Mouse Models Using ssGSEA. Heatmaps showing single‐sample gene set enrichment analysis (ssGSEA) scores for hallmark gene sets in mouse models of cardiomyopathy. Each panel represents one disease subtype: (A) ACM, (B) DCM, (C) HCM and (D) ICM. Rows correspond to annotated hallmark gene sets (left), and columns represent individual samples (top), grouped by disease versus wild type (WT). The colour scale ranges from blue (low ssGSEA score) to red (high ssGSEA score), reflecting pathway activity. This analysis validates the pathways identified through GSEA in human cardiomyopathy by assessing their enrichment in corresponding mouse models.

To validate our findings, we assessed the expression of key pathway changes and alterations in cell–cell communication associated with disease progression, using publicly available transcriptome data from preclinical mouse models, cell line models and patient samples representing each disease subtype. As shown in Figure 6A–D and Figure S4A–D, we observed that the disease‐enriched hallmark gene sets for each disease subtype, presented in Figure 3A, were consistently enriched in the transcriptome data from preclinical mouse models, cell line models and patient samples representing each disease subtype, respectively. For example, we observed the enrichment of the EMT signature across all four disease subtypes, as well as the enrichment of the IFN‐α response signature in DCM and HCM, which were consistent between both mouse and human sample data in the validated cohort. Notably, the prevalent enrichment of TGF‐β signalling across all disease subtypes, along with the angiogenesis signature closely linked to integrins, was also observed in all disease types. These findings further support and consolidate our conclusions on the role of cell–cell communication in disease progression.

However, due to data sparsity, the current datasets do not effectively characterise less abundant cell‐type populations, such as ILCs, which may be linked to chronic inflammation during disease progression. Future analyses could benefit from targeting these minority cell populations through novel techniques or increased sequencing depth.

These findings highlight the shared and unique cell–cell communication in each cardiomyopathy subtype, particularly the pathways identified through ligand‐receptor interaction analysis. These pathways reflect critical intercellular interactions within the heart, indicating both shared and unique physiological mechanisms that influence the pathogenesis of the studied cardiomyopathy subtypes. Future directions include leveraging multivariate analysis and machine learning to compare the importance of molecular features and other risk factors in disease progression, further aiding in the development of clinically applicable insights.

## Author Contributions

**Wenqi Tao:** conceptualization (supporting), formal analysis (equal), investigation (equal), methodology (equal), validation (equal), visualization (equal), writing – original draft (equal), writing – review and editing (equal). **Miao Gong:** formal analysis (supporting), project administration (supporting), writing – original draft (supporting), writing – review and editing (supporting). **Zunping Ke:** conceptualization (lead), formal analysis (equal), funding acquisition (lead), investigation (lead), methodology (lead), project administration (lead), supervision (lead), writing – review and editing (lead).

## Conflicts of Interest

The authors declare no conflicts of interest.

## Supporting information


Figure S1.

Figure S2.

Figure S3.

Figure S4.

Table S1.

## Data Availability

The data that support the findings of this study are openly available in https://cellxgene.cziscience.com/collections/e75342a8 0f3b 4ec5 8ee1 245a23e0f7cb/private; SCP1303 from Broad Institute Single Cell Portal; SCP1849 from Broad Institute Single Cell Portal. GSE101301, GSE106201, GSE112055, GSE129134, GSE289628, GSE116250, and GSE89714 are from GEO Data Sets: https://www.ncbi.nlm.nih.gov/gds/.
