# Supplementary material for: Characterising Shared and Specific Cell–Cell Communication in Cardiomyopathy Subtypes From Single‐Cell Transcriptomics Data
Source: J Cell Mol Med. 2025 May 8;29(9):e70554. doi: 10.1111/jcmm.70554 (PMC12061637; doi:10.1111/jcmm.70554)
Supplement: Supplementary file 1 — Figure S1. [file JCMM-29-e70554-s005.pdf]

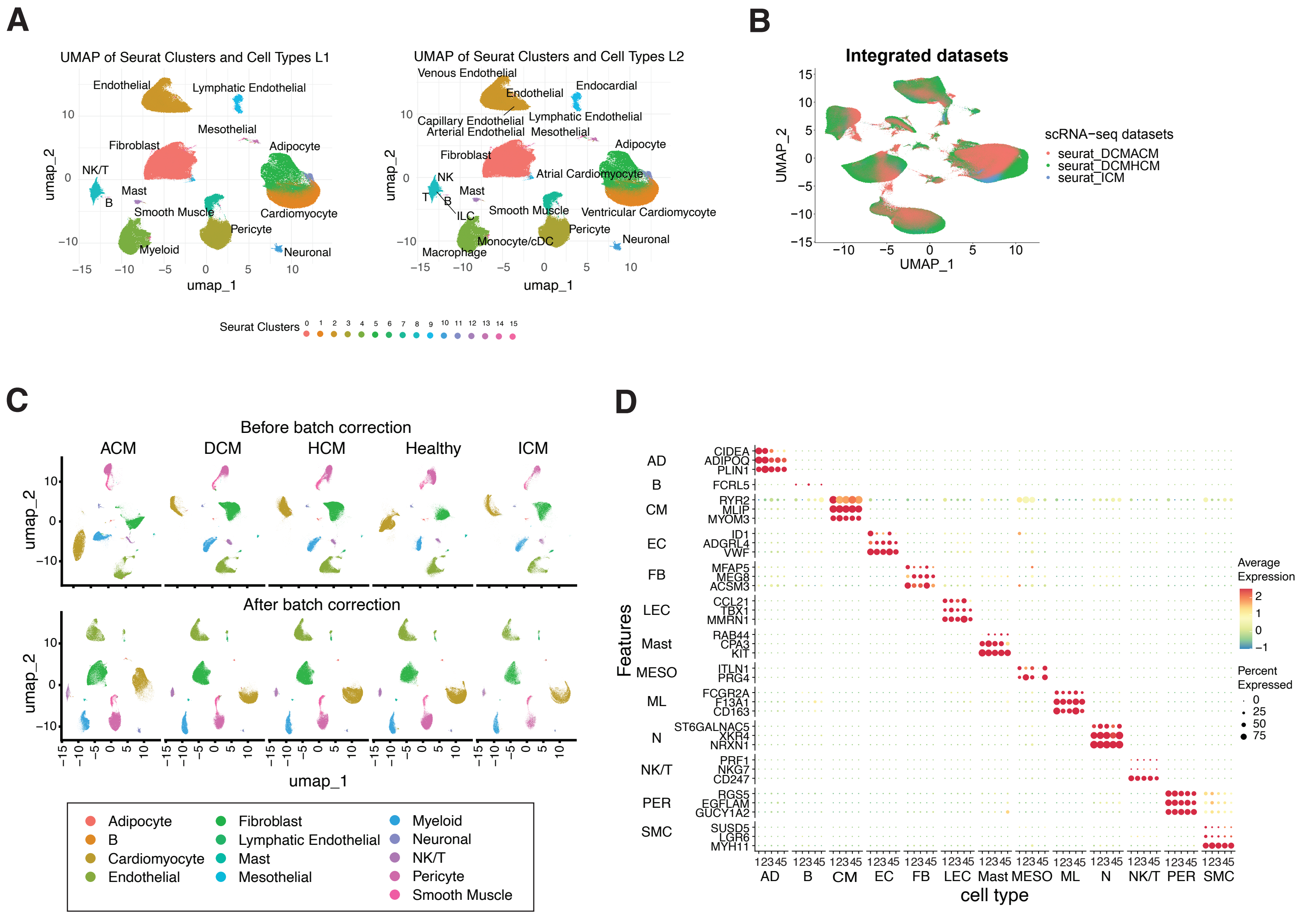

Figure S1: Identification of Major Cell Compartments in Human Cardiomyopathy and Healthy Donors through Single-cell Data Integration.

(A) UMAPs embeddings of 427,256 human heart ventricular cells following the merging and batch correction of samples from three datasets. Cells are colored by clusters identified using the Seurat 'FindClusters' function with default parameters. Cell types were annotated based on 'celltype.l1' (left panel) and 'celltype.l2' (right panel) using reference annotations from the Azimuth dataset. (B) UMAP visualization of 427,256 cells colored by batch origin. (C) UMAPs before (top panel) and after batch correction using Harmony (bottom panel), split by disease subtypes and healthy donors. Dot colors represent cell types annotated based on 'celltype.l1'. (D) Dot plot of marker genes (y-axis) across annotated cell types, shown by cell types and across each disease subtype and healthy donor group (x-axis). Dot size represents the portion of cells expressing the gene per cell type.
