# Supplementary material for: Characterising Shared and Specific Cell–Cell Communication in Cardiomyopathy Subtypes From Single‐Cell Transcriptomics Data
Source: J Cell Mol Med. 2025 May 8;29(9):e70554. doi: 10.1111/jcmm.70554 (PMC12061637; doi:10.1111/jcmm.70554)
Supplement: Supplementary file 2 — Figure S2. [file JCMM-29-e70554-s004.pdf]

A

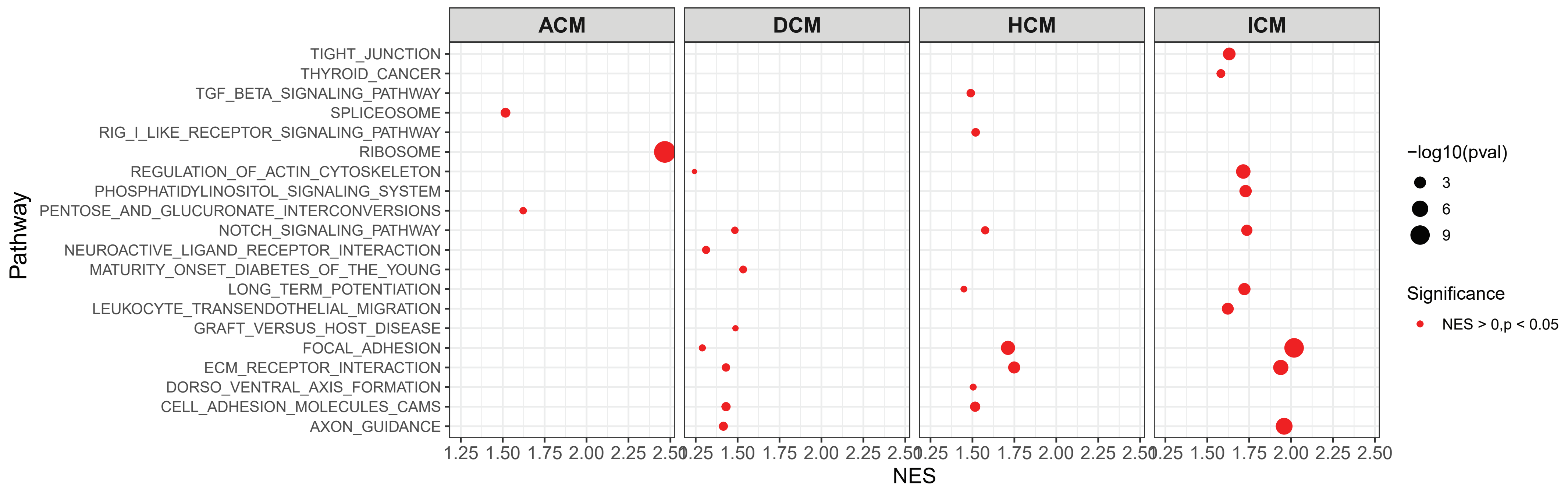

B

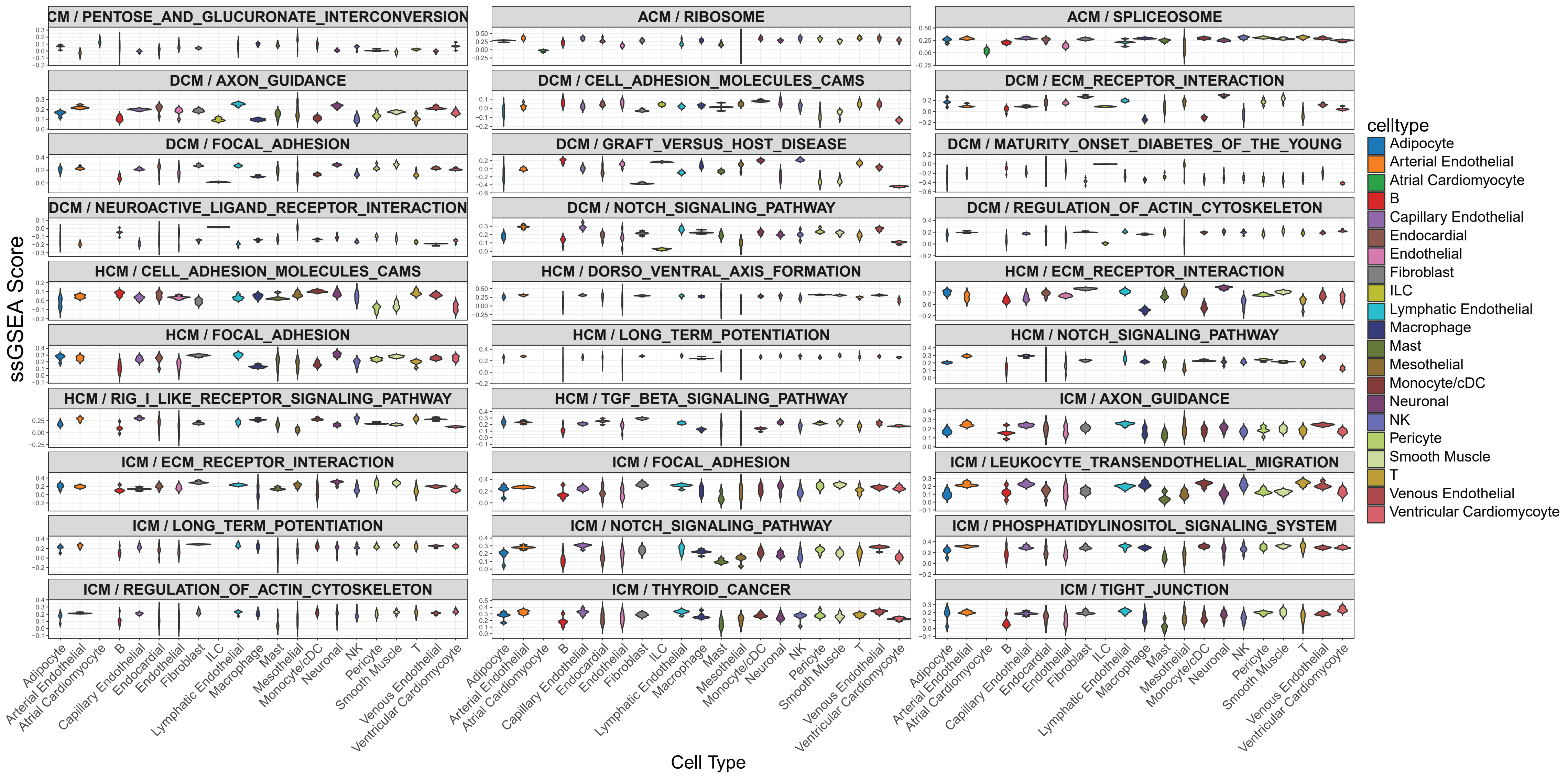

Figure S2. Gene Set Enrichment Analysis Reveals KEGG Gene Sets Across Cardiomyopathy Subtypes and Annotated Cell Types. (A) Dot plot showing MSigDB KEGG gene-set enrichment analysis comparing disease versus healthy controls across four cardiomyopathy subtypes (ACM, DCM, HCM, ICM). The plot is faceted by disease subtype, with the x-axis representing the normalized enrichment score (NES) and the y-axis indicating pathway names. Dot size corresponds to the -log10(p-value), and dot color indicates statistical significance. (B) Violin plot showing single-sample gene set enrichment analysis (ssGSEA) scores for pathways identified in Panel A across annotated cell types. The plot is faceted by enriched pathways from each disease subtype, with the x-axis and violin color representing the annotated cell types and the y-axis indicating the ssGSEA score.
