# Supplementary material for: Characterising Shared and Specific Cell–Cell Communication in Cardiomyopathy Subtypes From Single‐Cell Transcriptomics Data
Source: J Cell Mol Med. 2025 May 8;29(9):e70554. doi: 10.1111/jcmm.70554 (PMC12061637; doi:10.1111/jcmm.70554)
Supplement: Supplementary file 3 — Figure S3. [file JCMM-29-e70554-s003.pdf]

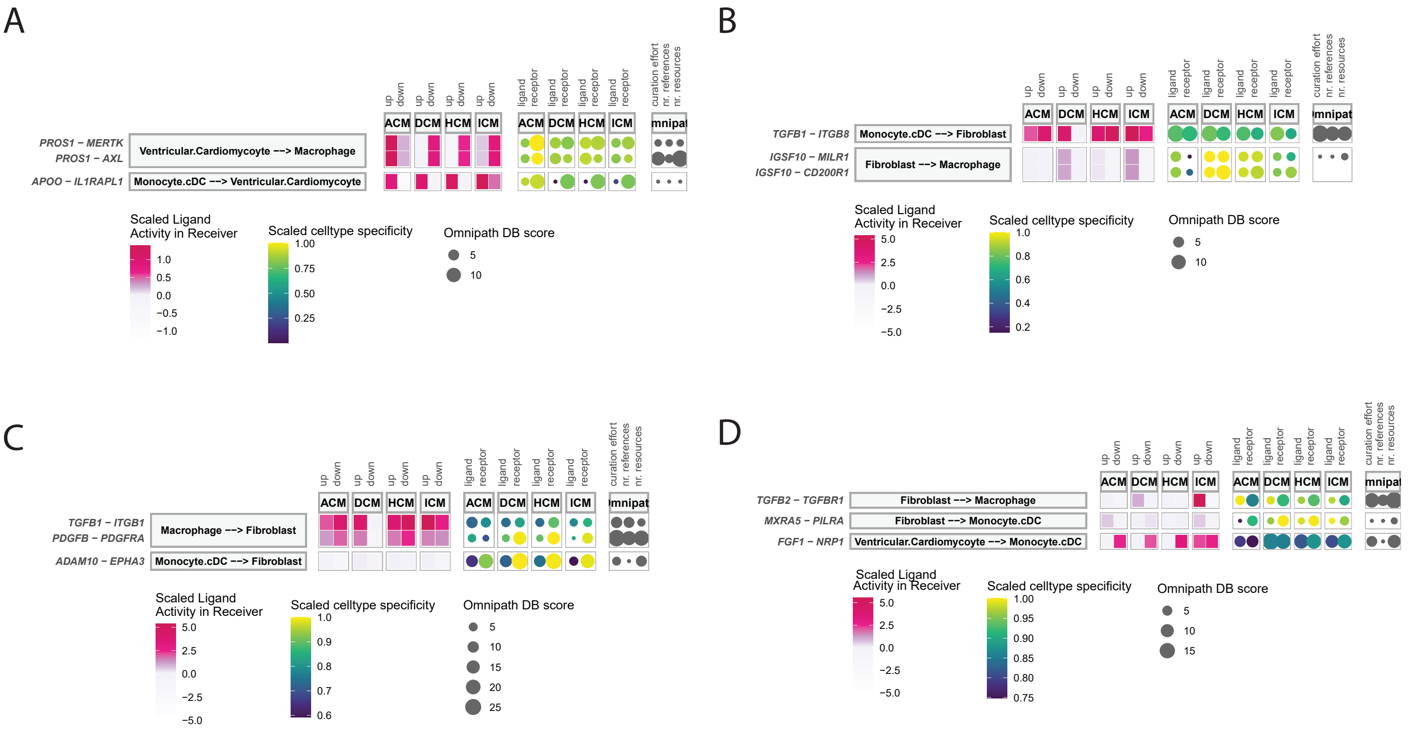

Figure S3. Summary of cell-cell communication in patients. Bubble plots summarizing the top three ligand-receptor (L-R) pairs between the most abundant immune cells (macrophages and Mono/cDCs) and the most abundant other cardiac cells (vCMs and FBs) for each cardiomyopathy subtype: (A) ACM, (B) DCM, (C) HCM, and (D) ICM. Interactome analysis was performed using the MultiNicheNet package on annotated scRNA-seq data comparing disease versus healthy controls. Each plot displays normalized ligand and receptor pseudobulk expression per sample, scaled ligand activity in receiver cells, scaled cell type activity, and curation levels of intercellular communication based on the Omnipath dataset.
