# Supplementary material for: Characterising Shared and Specific Cell–Cell Communication in Cardiomyopathy Subtypes From Single‐Cell Transcriptomics Data
Source: J Cell Mol Med. 2025 May 8;29(9):e70554. doi: 10.1111/jcmm.70554 (PMC12061637; doi:10.1111/jcmm.70554)
Supplement: Supplementary file 4 — Figure S4. [file JCMM-29-e70554-s001.pdf]

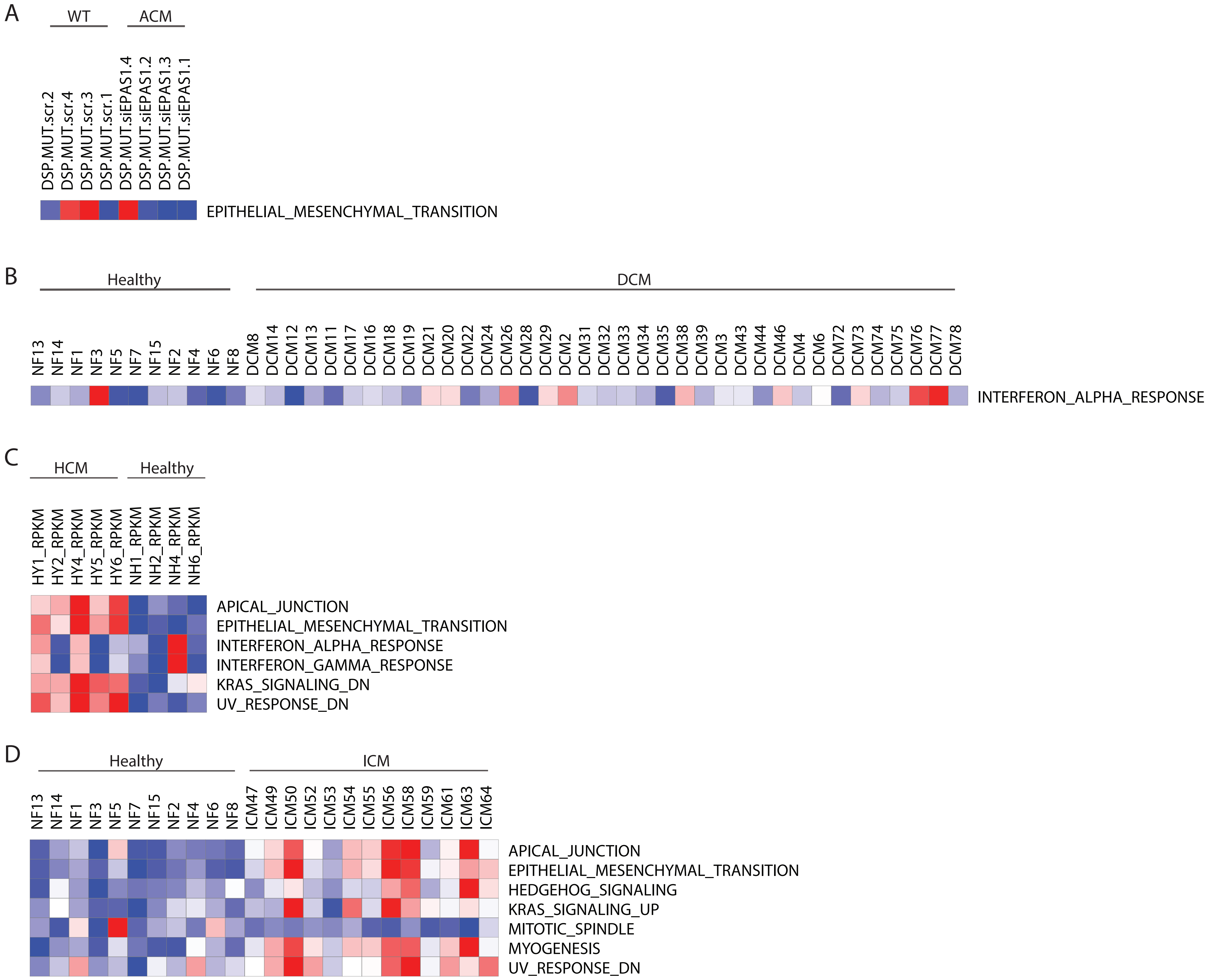

Figure S4. Validation of Disease-Enriched Pathways in Human Cell Lines and Patient Samples Using ssGSEA. Heatmaps showing single-sample gene set enrichment analysis (ssGSEA) scores for hallmark gene sets in human cardiomyopathy models. Each panel represents one dis-ease subtype: (A) ACM (human cell lines derived from hESCs), (B) DCM (patient samples), (C) HCM (patient samples), and (D) ICM (patient samples). Rows correspond to annotated hallmark gene sets (left), and columns represent individual samples (top), grouped by dis-ease versus wild type (WT). The color scale ranges from blue (low ssGSEA score) to red (high ssGSEA score), reflecting pathway activity.
